# Emotional Valence and the Free-Energy Principle

**DOI:** 10.1371/journal.pcbi.1003094

**Published:** 2013-06-13

**Authors:** Mateus Joffily, Giorgio Coricelli

**Affiliations:** 1 Center for Mind/Brain Sciences, University of Trento, Trento, Italy; 2 Groupe d'Analyse et de Theorie Economique, Centre National de la Recherche Scientifique, Lyon, France; 3 Department of Economics, University of Southern California, Los Angeles, California, United States of America; University of Oxford, United Kingdom

## Abstract

The free-energy principle has recently been proposed as a unified Bayesian account of perception, learning and action. Despite the inextricable link between emotion and cognition, emotion has not yet been formulated under this framework. A core concept that permeates many perspectives on emotion is valence, which broadly refers to the positive and negative character of emotion or some of its aspects. In the present paper, we propose a definition of emotional valence in terms of the negative rate of change of free-energy over time. If the second time-derivative of free-energy is taken into account, the dynamics of basic forms of emotion such as happiness, unhappiness, hope, fear, disappointment and relief can be explained. In this formulation, an important function of emotional valence turns out to regulate the learning rate of the causes of sensory inputs. When sensations increasingly violate the agent's expectations, valence is negative and increases the learning rate. Conversely, when sensations increasingly fulfil the agent's expectations, valence is positive and decreases the learning rate. This dynamic interaction between emotional valence and learning rate highlights the crucial role played by emotions in biological agents' adaptation to unexpected changes in their world.

## Author Summary

Emotion plays a crucial role in the adaptation of humans and other animals to changes in their world. Nevertheless, emotion has been neglected in Bayesian models of learning in non-stationary environments. The free-energy principle has recently been proposed as a unified account of learning, perception and action in biological agents. In this paper, we propose a formal definition of emotional valence (i.e., the positive and negative character of emotion) in terms of the rate of change of free-energy or, under some simplifying assumptions, of prediction error over time. This formalization leads to a straightforward and simple meta-learning scheme that accounts for the complex and reciprocal interaction between cognition and emotion. We instantiate this scheme with an emotional agent who is able to dynamically assign emotional valence to every new state of the world that is visited and to experience basic forms of emotion. Crucially, our hypothetical agent uses emotional valence to dynamically adapt to unexpected changes in the world. The proposed scheme is very general in the sense that it is not tied to any particular generative model of sensory inputs.

## Introduction

Free-energy is an information theoretic quantity that upper bounds the surprise on sampling some data, given a generative model of how data were caused. The free-energy principle assumes that biological agents encode a probabilistic model of the causes of their sensations, and postulates that any adaptive agent that resists a tendency to disorder must minimize its free-energy [1], [2]. Under simplifying (Gaussian) assumptions, free-energy minimization can be understood as the minimization of the prediction error between the actual and predicted sensory inputs. In order to comply with the free-energy principle, adaptive agents have two tactics at their disposal: (1) adjusting their internal states to generate more accurate predictions and (2) acting on the environment to sample sensations that fulfil their predictions. The principle is based upon the realization that perceptual inference and learning [3], [4] and active inference [5], [6] rest on the same Bayesian scheme. Perceptual inference refers to inferring the states of the world causing sensory inputs. Perceptual learning relates to learning the relationship between inputs and causes. Active inference corresponds to acting on the world to fulfil prior expectations about sensory inputs. The computational implementation of the free-energy principle has been shown to replicate in many aspects neural mechanisms and the cortical organization of the brain [4], [7].

Crucially, when inferring and learning the causes of their sensations in a changing world, adaptive agents need to deal with different forms of uncertainty, namely estimation uncertainty [8], volatility [9], [10] and unexpected uncertainty [11], [12]. Estimation uncertainty refers to the known estimation variance of states of the world causing sensory inputs and can be reduced through learning. Volatility refers to slow and continuous changes in states of the world, and has usually been modelled by making estimation uncertainty follow some latent stochastic process [13]. Finally, unexpected uncertainty arises from surprising sensory inputs caused by discrete and fast changes in states of the world, and calls for forgetting the past and restarting learning from new sensory data. Dealing with different forms of uncertainty is fundamental to Bayesian models of learning in a non-stationary environment [14]. In fact, a critical challenge faced by many recent Bayesian schemes of human learning is how to dynamically update beliefs about states of the world in order to optimize predictions in a changing environment [9]–[12], [15]–[18].

Despite the major role attributed to emotions in influencing the content and the strength of the agent's beliefs and the resistance of these beliefs to modification [19], emotion has not been considered in - much less integrated into - these computational models. The concept of emotional valence, or simply valence, has been used among emotion researchers to refer to the positive and negative character of emotion or some of its aspects, including elicitors (events, objects), subjective experiences (feeling, affect) and expressive behaviours (facial, bodily, verbal) [20]. The valence of feelings has been argued to be a pivotal criterion for demarcating emotion [21] and a core dimension of the subjective experience of moods and emotions [22]. Traditionally, mood has been defined, in contrast to emotion, as an affective state that lacks a clear referent, changes slowly and lasts for an extended period of time [23].

In the present paper, we propose a mathematical definition of emotional valence in terms of the negative rate of change of free-energy over time. As we shall see later, this formalism entails the dynamic attribution of emotional valence to every state of the world that an adaptive agent might visit and prescribes the dynamics of basic forms of emotion such as *happiness*, *unhappiness*, *hope*, *fear*, *disappointment* and *relief*.

We will first introduce the free-energy principle and present our computational model of emotional valence. We then demonstrate this scheme by simulating and comparing two artificial agents. One agent explicitly optimises posterior beliefs about volatility and does not use its internally generated emotional valence signal. The other does not estimate volatility but instead implements the emotional regulation of estimation uncertainty. The contribution of this work is two-fold. First, we provide a phenomenological account of emotion in terms of changes in free energy - as a proxy for changes in the quality of how the world is being modelled during inference and learning. Second, emotion is coupled back to inference using a form of regularization or meta-learning. In other words, changes in the quality of inference are used to regularize the rate of evidence accumulation to provide adaptive learning rates. These learning rates correspond to expected uncertainty about inferences, under hierarchical models of the world.

## Models

In this section, we introduce the free-energy principle as it was originally proposed by Friston, Kilner and Harrison [1], and then we propose a new mathematical definition of emotional valence and some basic forms of emotion in terms of free-energy. Next, we put forward a meta-learning rule by means of which emotional valence regulates estimation uncertainty, and outline the relationship between the dynamics of free-energy and some basic forms of emotion.

### The free-energy principle

In statistical physics, variational free-energy minimization is a method for approximating a complex probability density 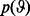 by a simpler ensemble density 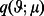 that is parametrized by adjustable parameters 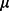 [24]. In neuroscience, the free-energy principle assumes that biological agents encode the parameters 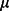 of an arbitrary recognition (ensemble) density 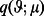 of environmental quantities 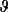 that are the presumed causes of their sensations [2]. The recognition density 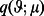 is an approximation to the true posterior density 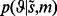 of 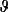, given the sampling of some sensory data 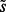 and the generative model 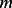 entailed by the agent.

The environmental quantities 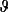 may be any forces or fields that act upon the agent, such as heat or light-stimulating sensory receptors. In more complex agents, 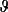 may also refer to very abstract quantities such as ‘social rank’ or ‘moral norms’. The learning of the environmental quantities 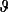 and inferences about their states rest on empirical Bayes and hierarchical generative models [3], [4]. In this framework, perceptual learning corresponds to estimating the parameters 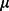 of the recognition density 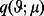 after many sensations, whereas perceptual inference corresponds to inferring the state of 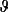 after a single sensation. In a hypothetical environment, learning could refer to the estimation of the categories associated with sensations while inference would be the classification of a particular sensation into one of these categories. In what follows, we shall see how free-energy minimization can account in a unified way for perception, learning and action.

The divergence from the recognition density 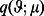 to the true posterior density 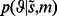 is measured by the Kullback-Leibler (KL) divergence, which can be decomposed into two quantities known as free-energy and surprise:
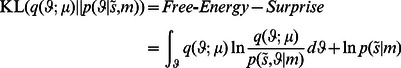
(1)


The first term on the right side of the equation is the free-energy that may be efficiently treated by adjusting the parameters 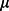 in order to minimize the divergence. The second term is surprise, which informs about the probability that some data 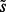 has been generated under the assumptions of the model 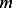. In Bayesian model selection, the marginal likelihood 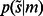 is also known as the evidence for the model 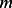. Rearranging (1), one obtains a formulation of free-energy 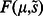 in terms of divergence plus surprise:
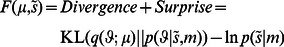
(2)


The free-energy principle states that any adaptive agent that is at equilibrium with its environment must minimize its free-energy [1], [2]. Minimizing free-energy with respect to 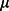 reduces divergence, thereby making the recognition density 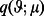 an approximate posterior density 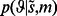. Notice that divergence depends on 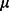 while surprise does not. Because the divergence is always non-negative (Gibb's inequality), free-energy is said to be an upper bound on surprise.

Crucially, biological agents can minimize free-energy not only by changing their beliefs but also by changing their sensations through acting on the environment. The dependency of sensation 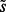 on action 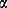 is expressed by 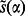. A new rearrangement of (1) shows more clearly what acting on the environment to minimize free-energy 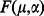 implies (here, we replace the dependency of free-energy on sensation 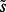 by expressing it directly as a function of 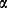):
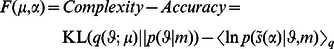
(3)where 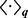 means the expectation under the density 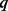.

In this second formulation, free-energy is expressed as complexity minus accuracy. Complexity is the divergence from the recognition density 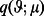 to the true prior density 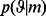 of the causes 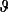. Accuracy is the surprise about sensations that are expected under the recognition density; note that accuracy depends on action 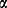 whereas complexity does not. This means biological agents will act to minimise free-energy through maximising accuracy. That is, biological agents will act in the environment to sample sensations that are expected by their recognition density.

This perspective on behaviour contrasts with the traditional one in Pavlovian and instrumental conditioning, where behaviour is chiefly understood in terms of maximizing expected reward or pleasure (or conversely minimizing expected loss or pain) [25], [26]. In active inference, behaviour is driven by an attempt to fulfil the sensory expectations of posterior beliefs (recognition density). This prevents the dichotomization of the states of the world in terms of pairs of opposites, such as ‘reward-loss’ or ‘pleasure-pain’, and implies that the notion of desired states is replaced with that of expected states. States with high probability under the recognition density (low surprise) are more frequently approached whereas states with low probability (high surprise) are avoided by the agent. Agents that expect to visit states that are noxious to their structure will compromise their chances of survival and transmitting their phenotype to future generations (e.g., a rabbit that expects to visit foxes). The adaptive fitness of a phenotype is thus the negative surprise averaged over all the states the agent visits [2].

### Emotional valence

In order to harmonize the principled assumption that any biological agent that is at equilibrium with its environment must minimize its free-energy [2] and the traditional notion that humans approach pleasure and avoid pain [27], we related positive and negative valence to the decrease and increase of free-energy over time, respectively. In a continuous time domain, the rate of change of free-energy 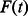 is the first time-derivative of free-energy 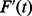 at time 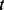. We thus formally define the valence of a state visited by an agent at time 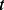 as the negative first time-derivative of free-energy at that state or, simply, 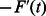.

Here, we recall that adaptive agents encode a hierarchical generative model of the causes of their sensations [3], [4]. States of the world of increasing complexity and abstraction are encoded in higher levels of the hierarchy, whereas sensory data *per se* are encoded at the lowest level. Free-energy is minimized for each level of the hierarchy separately, and the quantity 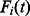 corresponds to the free-energy associated with the hidden state at the *i*-th level of the hierarchical model.

According to our definition of emotional valence, when 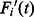 is positive (i.e., free-energy is increasing over time at level *i* of the hierarchy) the valence of the state at this level *i* is negative at time 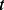. When 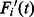 is negative (i.e., free-energy is decreasing over time at level *i*) the valence of the state at this level *i* is positive at time 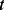. When 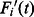 is zero (i.e., free-energy is constant at level *i*) the valence of the state at this level *i* is neutral at time 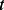. Importantly, free-energy is an upper bound on surprise, and neutral valenced states may also be characterized by low or high levels of surprise.

The factorization of emotional valence across levels of the hierarchical model means that positive and negative valence can be independently attributed to each state in the model, and thus positive and negative valences can be concurrently elicited for the same sensation. Note that free-energy and the rate of change of free-energy are functions not just of current sensations but the posterior beliefs about the causes of those sensations. This means that the free-energy can change in a way that is context-sensitive, depending upon (different) current beliefs about (exactly the same) sensations.

### Basic forms of emotion

Cognitive theories of emotion have widely relied on degrees of belief about states of affairs (environmental states) for their analyses of some basic forms of emotion. It has been suggested that a large group of emotions, which includes *happiness*, *unhappiness*, *relief* and *disappointment*, is related to certain (firm) beliefs that states of affairs obtain, while a second smaller group of emotions, mainly represented by *hope* and *fear*, is related to uncertain beliefs [28]–[31]. These two classes of emotions have been referred to as *factive* and *epistemic*, respectively [29]. In philosophy, states of affairs are formally said to either obtain or not whereas beliefs can be true or false (see [32]). Henceforth, we will adopt this terminology.

To illustrate the difference between *factive* and *epistemic* emotions, imagine the case of Lucia who is waiting for a train at the station. Lucia is *happy* that the train is on time (state of affairs 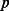), if she desires 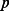 and is certain (i.e., firmly believes) that 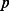 obtains. Conversely, Lucia is *unhappy* that 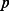, if she does not desire 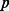 and is certain that 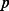 obtains. However, Lucia *hopes* that 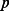, if she desires 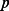 but is uncertain that 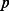 obtains; and, alternatively, Lucia *fears* that 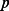, if she does not desire 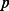 but is uncertain that 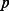 obtains. On the other hand, *relief* and *disappointment* are better related to the transition from uncertain to certain beliefs [31]. For instance, Lucia is *relieved* that *not-p* if she does not desire 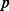 and up to now was uncertain about *p*, but now is certain that *not-p* obtains; conversely, Lucia is *disappointed* that *not-p* if she desires 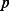 and up to now was uncertain about 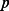, but now is certain that *not-p* obtains.

Beliefs and desires can be intuitively related to bottom-up conditional expectations and top-down predictions, respectively, in a predictive coding scheme of free-energy minimization [2]. In this formulation, states of affairs cannot be directly assessed but must be inferred from sensory inputs. Assigning absolute certainty (or zero uncertainty) to any belief impairs the learning of new relationships between sensory inputs and their causes. Here, we consider it more appropriate to circumvent the assumption of certain beliefs proposed in cognitive theories of factive and epistemic emotions, and present a new formulation that relies only on the dynamics of free-energy without any explicit reference to uncertainty. Later, we shall see that factive and epistemic emotions are indeed associated with low and high levels of uncertainty, respectively, but this comes as a consequence and not as a necessary condition of their definition (see Results).

In a continuous time domain, the rate of change of the first time-derivative of free-energy 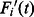 at the *i*-th level of the hierarchical model is the second time-derivative of free-energy 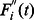. By analogy with mechanical physics, 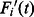 and 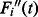 can be understood as the velocity and acceleration of free-energy 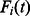 at time 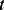, respectively. Our proposal stands on the assumption that, when both 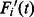 and 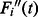 are negative (i.e., free-energy 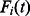 is decreasing ‘faster and faster’ over time) the agent *hopes* to be visiting a state of lower free-energy in the near future at this level *i*. However, when 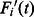 is negative and 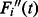 is positive (i.e., free-energy is decreasing ‘slower and slower’ over time) the agent is *happy* to be currently visiting a state of lower free-energy than the previous one at this level *i*. Equivalently, when 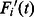 and 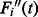 are positive (i.e., free-energy is increasing ‘faster and faster’ over time) the agent *fears* to be visiting a state of greater free-energy in the near future at this level *i*. However, when 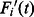 is positive and 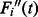 is negative (i.e., free-energy is increasing ‘slower and slower’ over time) the agent is *unhappy* to be currently visiting a state of higher free-energy than the previous one at this level *i*. Additionally, when the rate of change of free-energy 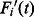 changes sign from negative to positive, the agent is *disappointed* not to be visiting a state of lower free-energy than the current one at this level *i*. Conversely, when 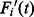 changes sign from positive to negative, the agent is *relieved* not to be visiting a state of higher free-energy than the current one at this level *i*. Finally, when 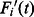 and 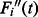 are zero (i.e., free-energy is constant over time) the agent may be low or high *neutrally surprised* in this level *i*. This analysis is summarized in Table 1. Note that since free-energy is minimized for each level of the hierarchical model separately, our formulation also predicts that different emotions can occur concurrently.

**Table 1:** Basic forms of emotion and the dynamics of free-energy.

Emotion at time 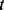	Valence	Factive/Epistemic	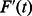 a	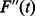 b
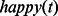	*positive*	*factive*	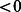	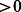
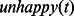	*negative*	*factive*	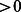	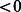
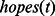	*positive*	*epistemic*	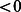	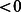
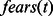	*negative*	*epistemic*	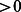	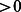
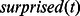	*neutral*	*factive*	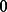	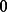
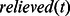	*positive*	*factive*	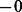 c	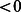
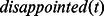	*negative*	*factive*	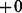 d	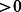

a First time-derivative of free-energy at time 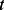.

b Second time-derivative of free-energy at time 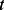.

c Negative value very close to zero.

d Positive value very close to zero.

**Figure 1:**
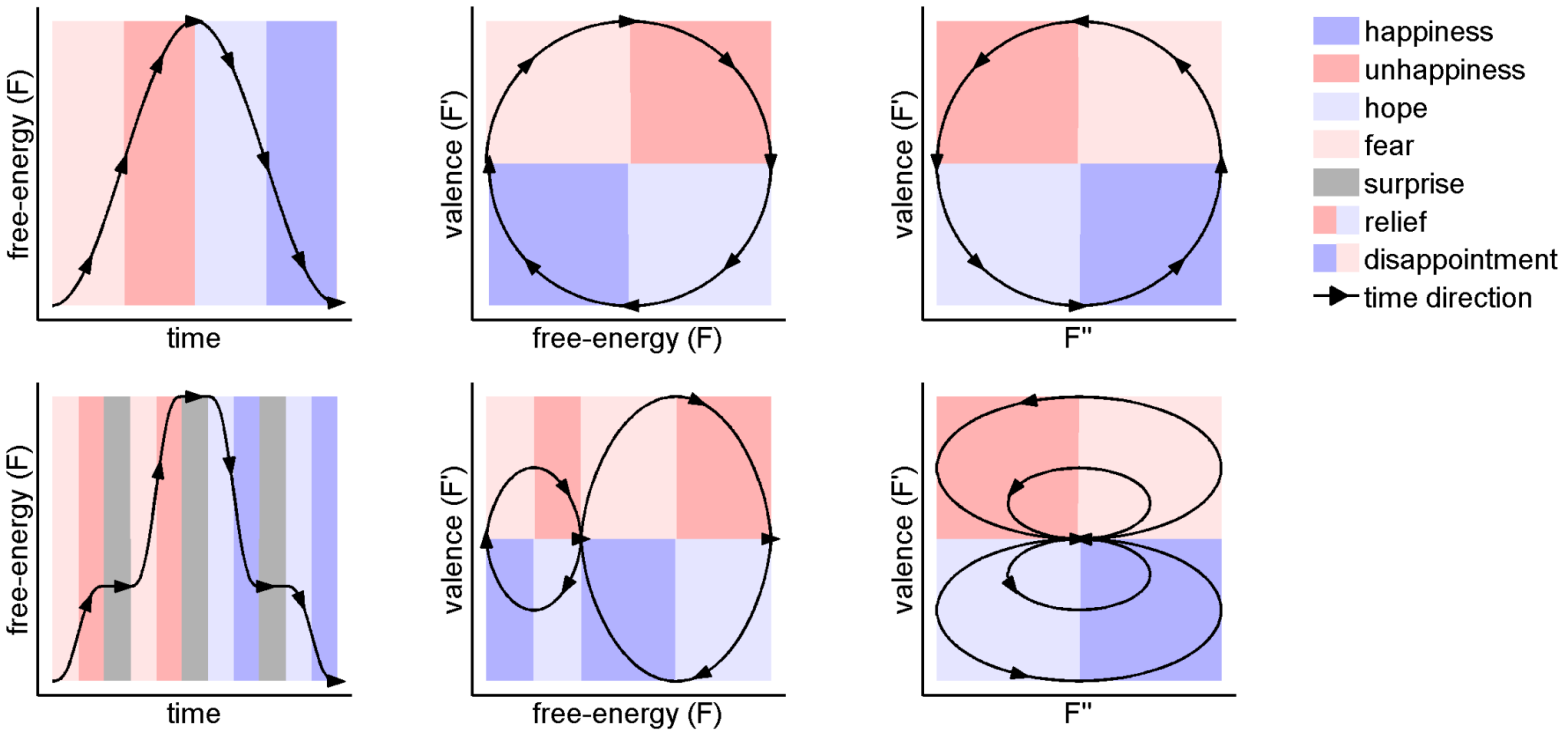
Basic forms of emotion and the dynamics of free-energy. (top and bottom rows) Two hypothetical dynamics of free-energy and their corresponding basic forms of emotion. (left column) Free-energy 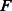 plotted as a function of time. (middle column) The same free-energy 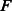 and its first time-derivative 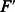 (valence) as a function of time. (right column) The first 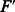 and second 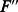 time-derivatives of the same trajectory of free-energy 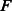 as a function of time. Notice that the basic forms of emotion are mapped to specific quadrants in the first and second time-derivative spaces independently of the free-energy trajectory. The black arrows indicate the direction of increasing time. The background colours identify the basic forms of emotion elicited at each time point: *happiness* (dark blue), *unhappiness* (dark red), *hope* (light blue), *fear* (light red), *relief* (transition from dark red to light blue), *disappointment* (transition from dark blue to light red) and *surprise* (grey).

The dynamics of free-energy reveal an interesting temporal dependency among the basic forms of emotion. Figure 1 illustrates two hypothetical dynamics of free-energy (top and bottom rows) that elicit distinct patterns of emotion over time (left column). From the two-dimensional space defined by the first and second time-derivatives of free-energy (right column), it becomes clear that transitions from negative to positive emotions can only occur by passing through *relief*, and transitions from positive to negative emotions can only occur by passing through *disappointment*, but transitions between negative (e.g., *fear* and *unhappiness*) or positive (e.g., *hope* and *happiness*) emotions can occur bidirectionally. More importantly, each basic form of emotion is mapped onto a particular region of this two-dimensional space.

### Emotional regulation of estimation uncertainty

So far, we have described how emotional valence and some basic forms of emotion can be elicited by the dynamics of free-energy. What, however, is the function of these quantities in a scheme originally developed to explain perception, learning and action? We propose that valence, computed as the negative rate of change of free-energy, is crucial because it informs biological agents about unexpected changes in their world. When valence is positive, sensory inputs fulfil the agent's expectations and the probability of unexpected changes is low. However, when valence is negative, the agent's expectations are violated and unexpected changes in the world are likely to have taken place. In settings where recent information is a better predictor of states of the world than past information, that is, in a changing world, recent information must be more heavily weighted and, therefore, the learning rate should be high [14]. Conversely, in a stationary world, in which past and recent information are equally informative, the learning rate should be low in order to take into account both past and recent information.

We formalise this notion in terms of emotional meta-learning in which estimation uncertainty is determined not just by free-energy but by the rate of change of free-energy. More specifically, when the free-energy associated with posterior beliefs about states at a particular level in the agent's hierarchical model is increasing, the posterior certainty about these states decreases. In other words, the agent interprets decreasing evidence for its estimates of states of the world as evidence that it is too confident about those states. This can be implemented fairly simply with the augmented Bayesian update:
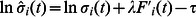
(4)

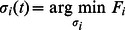
(5)

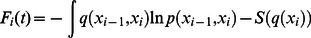
(6)


Here, the variances 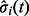 and 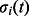 correspond to the posterior estimation uncertainty with and without emotional regulation, respectively. The variance 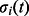 is the one that changes to minimize the free-energy 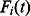 at the *i*-th level of the generative model. The quantity 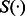 denotes the Shannon entropy, which in this case is a measure of the uncertainty associated with the states at level *i* in the recognition density. The parameter 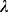 can be interpreted as the sensitiveness or ‘awareness’ of the agent to its emotional valence signals, which informs the agent about changes in the world. The parameter 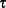 represents a long-lasting valenced level that lacks a clear referent, which we thus interpret as mood [23]. The parameters 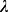 and 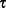 are both state and agent dependent. They can also be interpreted as the agent's meta-cognition about the extent to which the agent knows that it does not know the structure of the world.

We have framed the emotional regulation of uncertainty as meta-learning to emphasise that learning (the update) is informed by the consequences of learning, here, the rate of change of variational free-energy. Note that this is a very general scheme that is not tied to any particular generative model. Crucially, expectations about various states, which define them as surprising or not, rest upon prior beliefs that are themselves optimised with respect to variational free-energy; either at an evolutionary timescale or during experience dependent learning.

From equation 4, one can see that positive and negative valence exponentially decreases and increases, respectively, the estimation uncertainty about states of the world. The mood 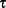 induces a constant level of over or under-confidence in the estimates of states irrespective of how surprising the sensory inputs may be. In a negative mood (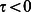), the agent overweights recent inputs, tracking more easily any volatility in the environment. In a positive mood (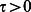), the agent overweight past inputs, becoming more attached to past information and less susceptible to tracking environmental changes.

This emotionally regularized update scheme may appear a bit ad hoc. However, there are several important heuristics in the optimisation literature that are closely related to Equation 4. These are generally described as regularization schemes - for example Levenberg Marquardt Regularization - in which the gradient descent or learning rate is generally decreased when the objective function being optimized does not change as expected. Usually, this regularization can be cast as changing the relative precision of the data at hand. In short, like our scheme, regularization schemes detect a failure in optimization in terms of adverse changes in the objective function (here the free energy) and respond by making more cautious updates - through changing the expected uncertainty about data or prior beliefs. We will see later that, in a hierarchical setting, this can lead to an adaptive change in the rate of optimization or learning at various levels of a hierarchical model.

## Results

In this section, we apply equation 4 to demonstrate how one can simulate an emotional agent. In brief, we will compare and contrast two schemes that are exposed to exactly the same sensory inputs and do or do not have emotional updates. The first is based on a hierarchical Bayesian treatment of volatility models that explicitly optimises posterior beliefs about estimation uncertainty. The second uses a simpler generative model that does not optimise the estimates of uncertainty explicitly but implements valence. Using this simpler scheme we show that the same adaptive behaviour can be reproduced using the emotional updates above.

### The dynamic perceptual model

Mathys et al. [10] have proposed a generic hierarchical Bayesian scheme that accounts for learning under multiple forms of uncertainty and environmental states. The environmental states can be either discrete or continuous, and the uncertainty can range from probabilistic relations between environmental and perceptual states (perceptual ambiguity) to environmental volatility. Here, we focus only on the simplest discrete and deterministic (i.e., without perceptual ambiguity) environment which nevertheless includes volatility.

In our example of a discrete and deterministic environment, we simulate an agent that learns the probability of a slot machine (one-armed bandit) to generate outcomes (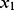) equal to either $1 (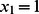) or $0 (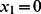). The agent's sensations (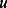) of the outcomes (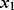) are unambiguous, meaning that 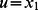 for both 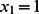 and 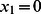. The reward probability of the slot machine is governed by the tendency (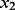) of the machine to generate $1. In the dynamic perceptual model, the agent knows that the reward tendency may change over time and therefore they also estimate its volatility (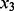).

This discrete and deterministic environment can be formalized with the statement that the sensory input 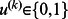 is binary and the environmental state 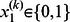 is the deterministic cause of input 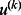 at trial 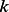. The likelihood of state 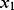 given sensory input 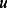 has the following form (for simplicity, we omit the trial reference 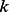):
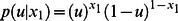
(7)


Therefore, 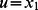 for both 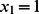 and 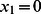. At the next level of the hierarchy, the tendency of 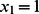 (i.e., outcome equal to $1) is defined by the state 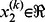. The probability of 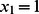 approaches zero when 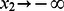 and approaches one when 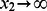. The mapping from the tendency 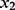 to the probability of 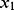 is defined by the following empirical (conditional) prior density:
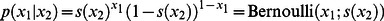
(8)where 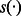 is the sigmoid function:
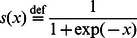
(9)


It is also assumed that the state 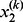 at trial 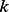 is normally distributed around its value at the previous trial 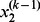 with variance 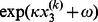. In other words, 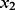 evolves in time as a Gaussian random walk:
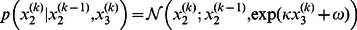
(10)where the parameters 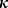 and 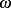 are agent dependent.

The state 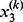 determines the log-volatility of the environment and is represented at the third level of the model. Again, 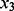 also evolves in time as a Gaussian random walk but with a step size defined by the constant 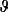 that may also differ among agents:
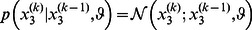
(11)


This structure defines a four-level generative model, where 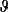 is represented at the last level, and its inversion corresponds to optimizing the posterior densities over unknown hidden states 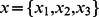 and parameters 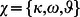. Here, states and parameters are distinguished in terms of the timescale at which they change. More specifically, states change quickly and parameters change either slowly or not at all for the duration of the observations.

### The static perceptual model with emotional valence

Alternatively, we propose a generative model that does not explicitly estimate the volatility (e.g., 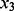) of some environmental states (e.g., 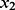) but instead makes use of emotional valence (i.e., the negative rate of change of free-energy over time) to assess unexpected changes in the environment. For that purpose, we implement the static perceptual model proposed by Daunizeau et al. [15] with two modifications. First, we consider unambiguous sensory inputs as in Mathys et al. [10] and, second, we use valence to update the posterior variance (estimation uncertainty) of states according to equation 4.

At the first level of the hierarchy, the dynamic model and static perceptual model with valence are exactly the same. At the second level, the static model assumes that the tendency 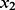 of outcome 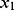 to be equal to $1 is constant across trials:
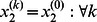
(12)


After inverting this generative model using variational free-energy minimization as described in [10], [15], we obtain the updated equations of the posterior distribution of 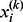, which can be used to investigate the behaviour of the agent on a trial-by-trial basis:
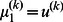
(13)

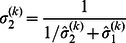
(14)

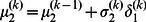
(15)where the following definitions have been used:
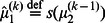
(16)

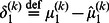
(17)

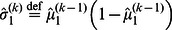
(18)

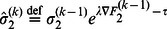
(19)


Here, 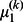 and 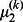 are the posterior expectations of 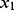 and 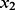 after sensory input 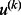, which can be interpreted as the expected probability and the expected tendency of reward, respectively. Accordingly, the uncertainty 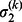 is the posterior variance of 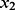. The prediction error at the first level 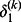 is the difference between the expectation 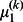 and the prediction 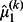 before seeing the input 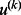. Equivalently, 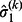 is the variance of the prediction 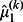 before seeing the input 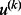.

In order to adapt to unexpected changes in the environment, the agent needs to update the posterior variance 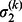 proportionally to the valence of the state 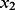 at time 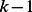. In discrete time, the valence of the state 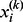 is, by definition, the negative first backward difference of free-energy 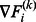 at time 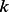:
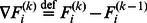
(20)


Specific to the proposed generative model, the free-energy 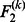 of state 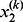 is:
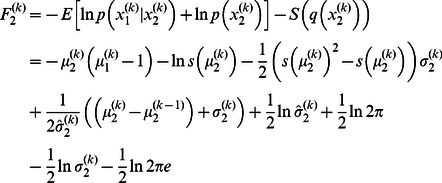
(21)where the expectation is taken under the approximate posterior densities 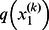 and 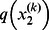.

The parameters 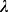 and 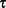 are constant and dependent on the agent. They represent the sensitiveness to emotional valence and the mood of the agent, respectively. According to our assumptions, the uncertainty of a hidden state 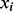 should increase or decrease when its valence 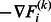 is negative or positive, respectively. Therefore, 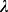 is constrained within the interval 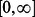. Notice that, when 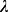 and 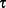 are equal to zero, the static perceptual model with valence becomes the same as the standard static perceptual model described in [15].

### The reference scenario

Having defined the two competing schemes, we implemented two agents under the dynamic perceptual model (DP) and the static perceptual model with valence (SPV), hereafter referred to as the DP agent and the SPV agent. These agents were exposed to 320 sensory inputs (outcomes) sampled from a three-stage reference scenario as proposed in [10]. In the first stage (low volatility) of the scenario, the agents were exposed to a sequence of 100 outcomes where the probability of 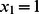 (outcome equal to $1) was 0.5. In the second stage (high volatility), the probability that 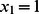 alternated between 0.9 and 0.1 every 20 inputs. Finally, in the third stage (low volatility again), the first 100 outcomes were repeated in exactly the same order. The initial values of the hidden states 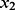 and 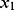 were 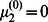, 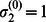 and 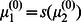 for both the DP and SPV models. In the DP model, the initial values of the hidden state 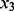 were 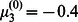 and 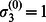.

**Figure 2:**
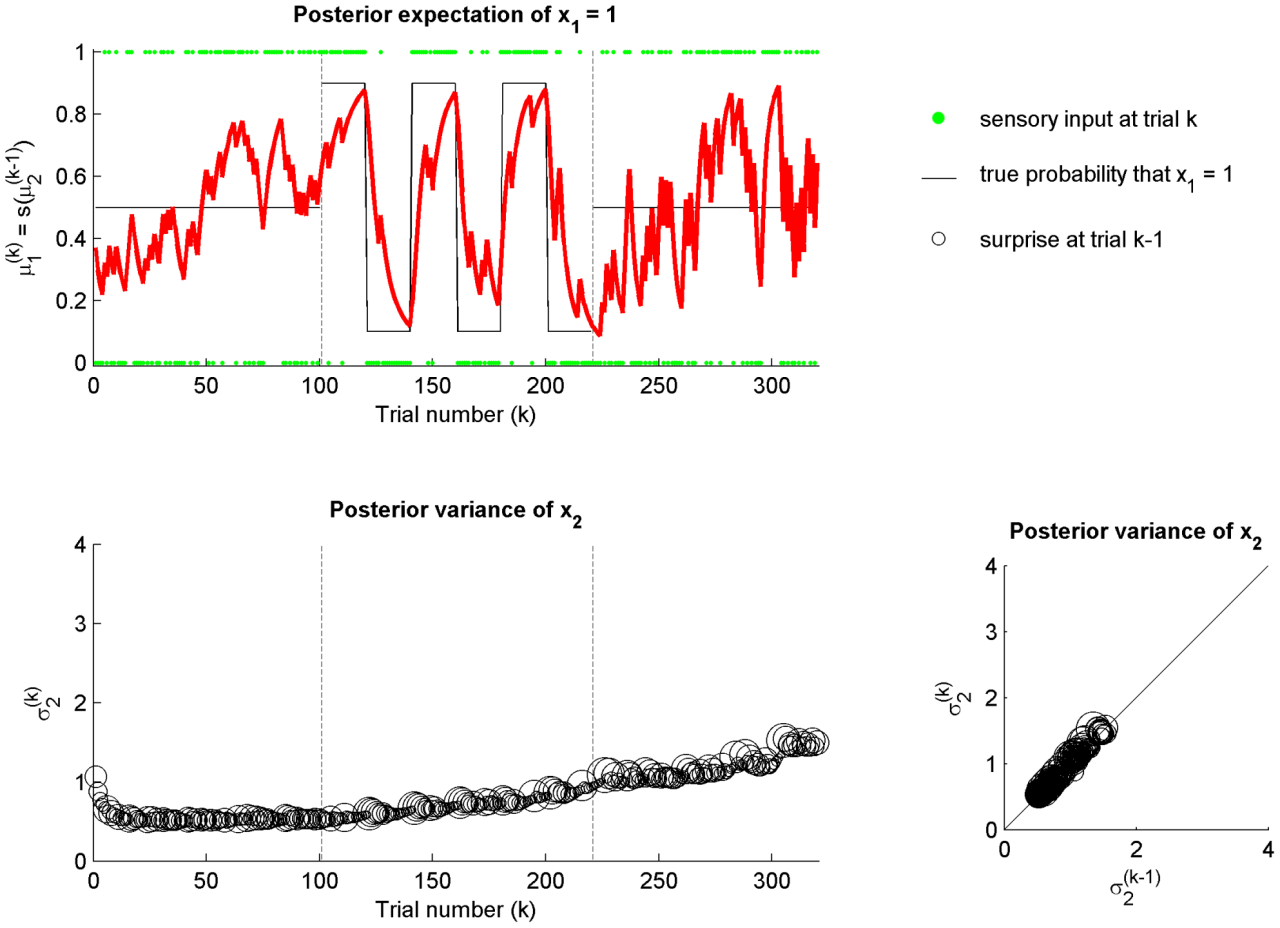
Dynamic perceptual model: 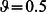, 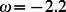 and 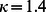. A simulation of 320 trials. The first (low volatility), second (high volatility) and third (low volatility) stages are separated by vertical dashed lines. (top) The agent's posterior expectation 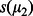 that 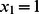 (red line) after sensory input 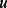 (green dots), is plotted over the true probability that 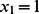 (black line), which is unknown to the agent. (bottom left) The time course of the posterior variance 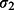 of 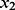 over trials. The size of the black circles is proportional to the surprise of sensory input 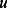 at trial 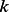. (bottom right) The change in the posterior variance of 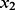 from trial 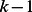 to trial 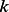 as a function of the surprise of sensory input 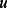 at trial 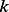.

We replicated the results reported by Mathys et al. [10] for the DP model with the same parameters 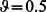, 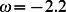 and 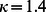 (see Figure 2). Overall, the posterior expectation of 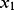, which is the reward probability, fluctuated around the true probability of 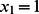 both in the low and high volatility stages. Nevertheless, one can observe increasing instability during the third stage relative to the first, even though the inputs were presented exactly in the same order in both of them. Mathys et al. [10] explained this in terms of a strong tendency for the agent to increase its posterior expectation of log-volatility 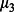 in response to surprising stimuli (given the parameters used in the reference scenario). The increase of 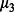 was followed by an increase in the posterior variance 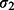 of state 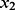, which regulates the learning rate at the second level. Despite the different levels of volatility in each stage, the posterior variance 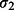 smoothly increased with a constant rate during the whole scenario.

**Figure 3:**
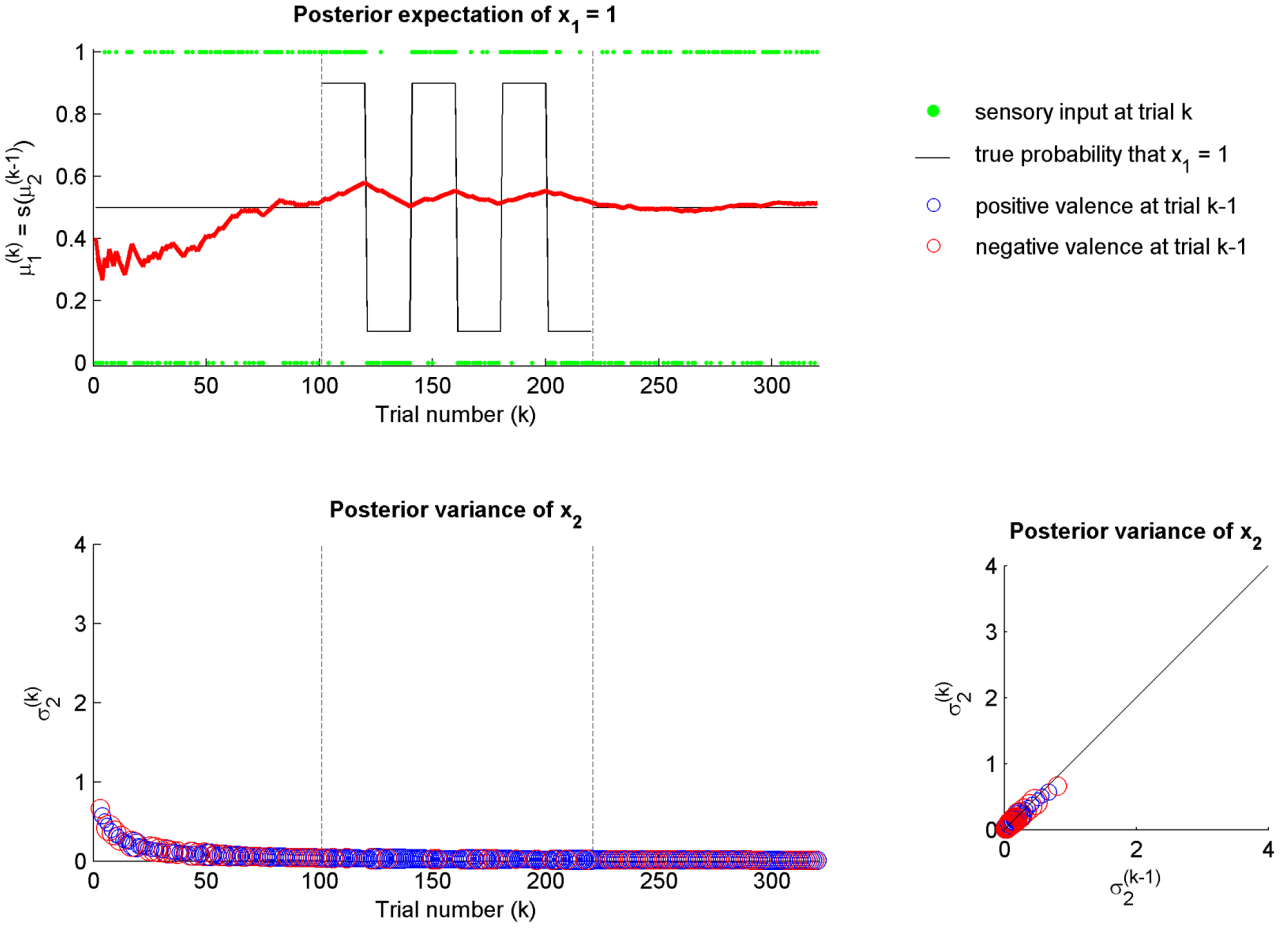
Static perceptual model: 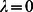 and 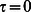. The agent is exposed to the same sequence of sensory inputs described in the reference scenario (see Figure 2 for legends). (top) The posterior expectation of 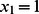 converges to 0.5, which is the true probability of 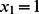 across the three (low and high volatility) stages. The agent is unaware of unexpected changes in the environment. (bottom left) The posterior variance (estimation uncertainty) 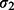 of 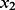 asymptotically converges to zero across trials 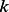. Negative (red circle) and positive (blue circle) valences are indicated when elicited over the trial. The size of the circles is proportional to the surprise of the sensory input 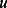 at trial 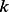. (bottom right) The change in the posterior variance of 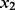 from trial 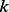 to trial 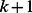 as a function of negative (red circle) and positive (blue circle) valences.

We first evaluated the SPV model setting both the sensitiveness 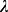 and mood 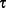 equal to zero. In this case, the agent learns according to a standard static perceptual model and is completely insensitive to any volatility or unexpected change in the environment. As illustrated in Figure 3, the posterior expectation of 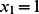 converges to 0.5, which is the true probability of 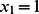 across the three (low and high volatility) stages. Concomitantly, the posterior variance (estimation uncertainty) 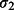 asymptotically decreases toward zero, reflecting the decreasing uncertainty of the estimates across sensory inputs.

**Figure 4:**
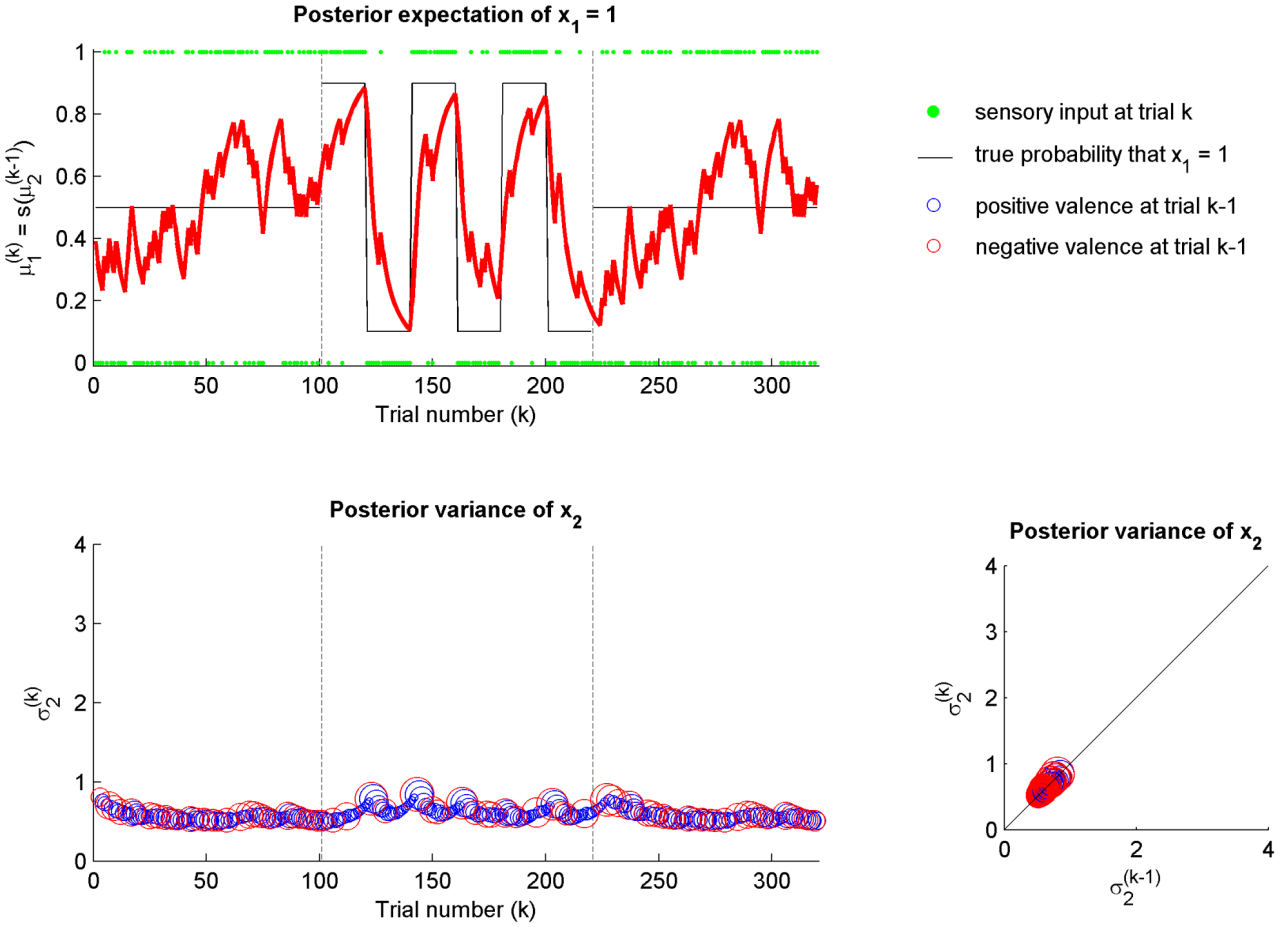
Static perceptual model with valence: 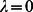 and 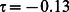. The agent is exposed to the same sequence of sensory inputs described in the reference scenario (see Figure 2 for legends). Now, the agent becomes reactive to unexpected changes in the environment. (top) The posterior expectation of 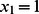 fluctuates around the true probability of 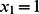 at each stage in a manner similar to the dynamic perceptual model (see Figure 2). (bottom left) The posterior variance (estimation uncertainty) 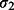 maintains a constant baseline during the first and third (low volatility) stages mainly defined by the mood, but starts to show a tendency to fluctuate more freely during the second (high volatility) stage. (bottom right) The change in the posterior variance of 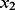 from trial 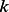 to trial 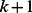 as a function of negative (red circle) and positive (blue circle) valences is quite similar to the standard static model (see Figure 3), except for a small offset defined by the mood.

When setting the parameters 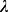 and 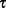 to values different than zero, the agent becomes sensitive to changes in its environment. In Figure 4, one can observe the effect of mood 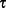 alone. When 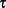 is set to −0.13 and 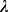 is kept equal to 0, a negative mood is sufficient to make the SPV model reactive to the volatility of the environment similar to the DP model. Importantly, the dynamic model also has a constant parameter 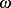 that is agent dependent, which has a similar function to 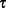 in our model. Nevertheless, the SPV does not show the increasing instability in the last (low volatility) stage observed in the DP model. In fact, the posterior variance 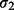 returns to a stable baseline even after the increased fluctuation during the high volatility stage.

**Figure 5:**
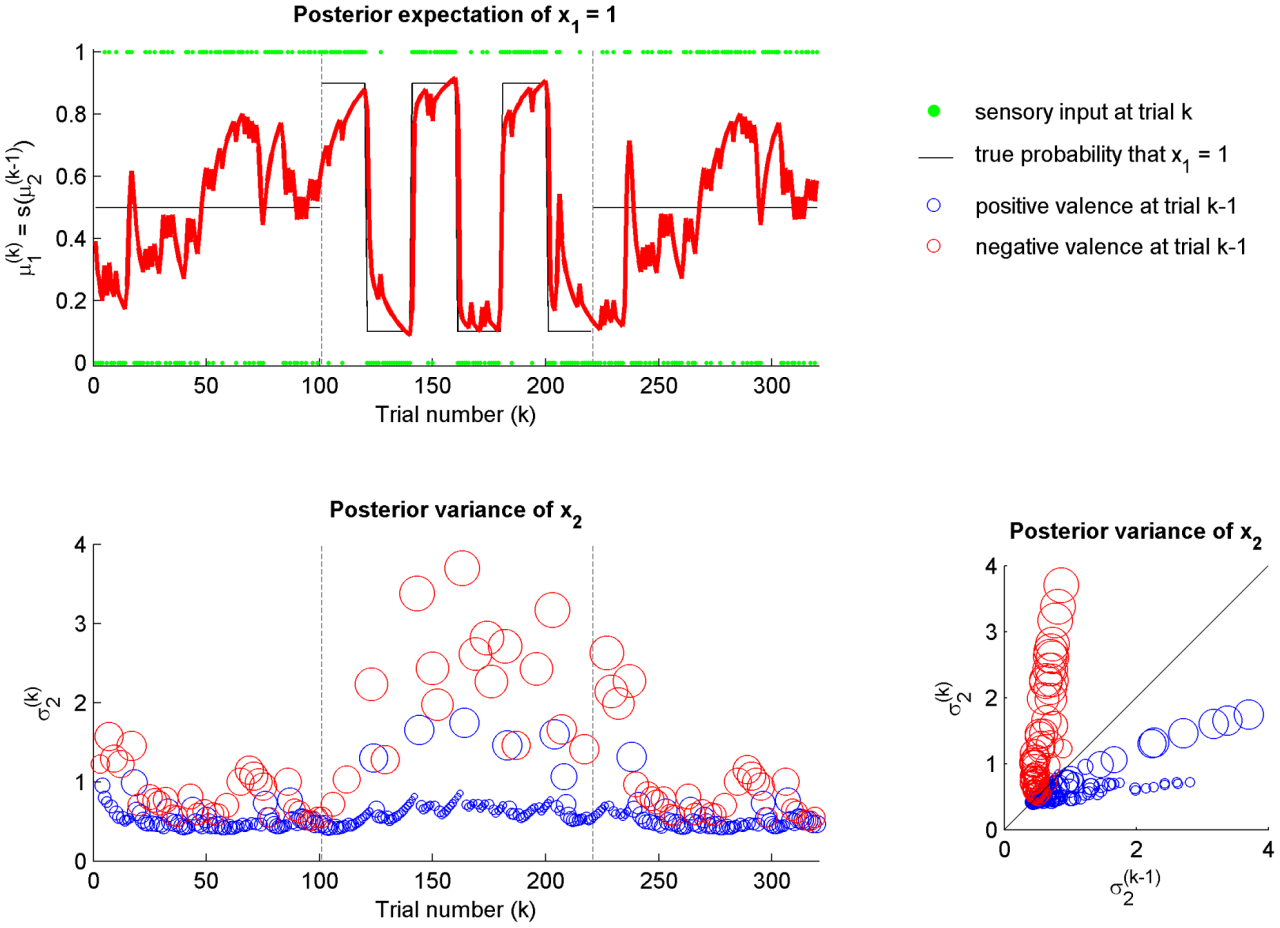
Static perceptual model with valence: 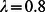 and 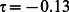
 **.** The agent is exposed to the same sequence of sensory inputs described in the reference scenario (see Figure 2 for legends). Now, the agent becomes extremely reactive to unexpected changes in the environment. (top) The posterior expectation of 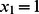 changes more quickly and is closer to the true probability of 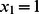 at each stage. (bottom left) The posterior variance (estimation uncertainty) 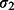 maintains a constant baseline during the first and third (low volatility) stages mainly defined by the mood, but it fluctuates more widely during the second (high volatility) stage. This clarifies the distinction between the low and high volatility stages. Negative (red circle) and positive (blue circle) valences are clearly associated with increases and decreases in uncertainty, respectively, and they become more intense during the second (high volatility) stage. (bottom right) The posterior variance of 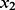 from trial 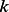 to trial 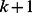 increases after negative valence but decreases after positive valence.

With the addition of emotional valence to the model, the agent becomes even more reactive and is able to track fast changes in the environment. In Figure 5, the sensitiveness 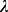 is set to 0.8. The posterior variance 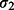 now changes more quickly in response to surprising sensory inputs and there is a clear distinction between the low and high volatility stages. More specifically, the elicitation of negative valence is the main cause of increases in 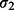, whereas positive valence causes 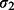 to decrease. Despite the phasic reaction to unexpected changes during the high volatility stage, the agent returns again to a fairly stable baseline similar to the first low volatility stage in the last low volatility stage.

Critically, an optimal tracking of environmental volatility requires mood to be set to some appropriate negative value. An extremely low mood, characterized by a large negative tau, would cause a very large increase in estimation uncertainty, consequently impairing discrimination between high and low volatility stages.

### Uncertainty associated with factive and epistemic emotions

We also investigated the estimation uncertainty associated with the *factive* (*happiness* or *unhappiness*) and *epistemic* (*fear* or *hope*) emotions in the reference scenario. It is noteworthy that we defined these emotions simply in terms of the dynamics of free-energy without any assumptions about uncertainty, contrary to the traditional analysis of these emotions in psychology and philosophy (see [28]–[31]). For this purpose, we performed 100 realizations of the reference scenario (i.e., we repeated the simulation with the reference scenario 100 times, sampling new sensory inputs at each time) and we computed the mean of the posterior variance (estimation uncertainty) 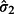 of state 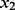 immediately after the onset of *factive* and *epistemic* emotions. The posterior variance 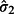 represents the change in estimation uncertainty after the elicitation of the emotion and before the observation of the next sensory input (see equation 19). For this analysis, we set the sensitiveness 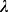 to an intermediate value equal to 0.4 and we kept the mood 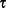 equal to −0.13.

**Figure 6:**
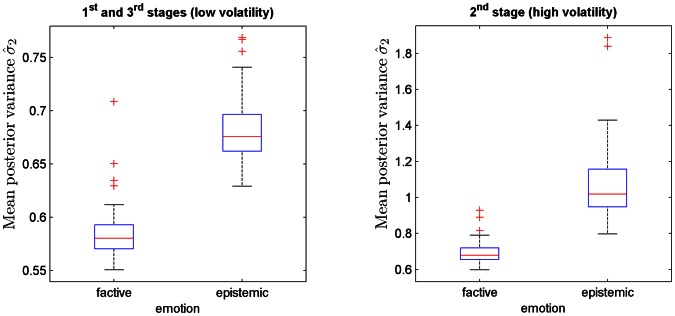
Boxplots of the mean posterior variance 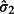 of state 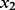 after the elicitation of *factive* (*happiness* or *unhappiness*) and *epistemic* (*fear* or *hope*) emotions and before the observation of the next sensory input. (left) Mean posterior variance 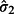 during the low volatility stages of the reference scenario. (right) Mean posterior variance 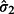 during the high volatility stages of the reference scenario. The mean 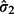 was computed for each of 100 simulations of the reference scenario. In both the low and high volatility stages, the mean 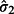 was on average higher for the *epistemic* (low volatility: M = 0.68, SD = 0.03; high volatility: M = 1.07, SD = 0.19) than the *factive* (low volatility: M = 0.58, SD = 0.02; high volatility: M = 0.69, SD = 0.06) emotions and it was also on average higher during the high (M = 0.88, SD = 0.24) than the low volatility (M = 0.63, SD = 0.06) stages.

The distribution of the mean 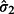 across simulations grouped within the low and high volatility stages of the reference scenario is shown in Figure 6. In both the low and high volatility stages, the mean 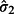 was higher on average for the *epistemic* (low volatility: M = 0.68, SD = 0.03; high volatility: M = 1.07, SD = 0.19) than the *factive* (low volatility: M = 0.58, SD = 0.02; high volatility: M = 0.69, SD = 0.06) emotions. Furthermore, the mean 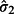 was also higher on average during the high (M = 0.88, SD = 0.24) than the low volatility (M = 0.63, SD = 0.06) stages.

## Discussion

In this paper, we have proposed a biologically plausible computational model of emotional valence inspired by the free-energy principle. The mathematical definition of emotional valence in terms of the negative rate of change of free-energy not only accounts for how beliefs determine emotions but also provides a formal account of how emotions determine the content and the degree of posterior beliefs (see [19]). In our framework emotional valence regulates estimation uncertainty signalling unexpected changes in the world, thereby performing an important meta-learning function.

The relationship between emotional valence and state transition also finds support in previous studies of emotion (see [33]–[36]). Batson et al. [35] have argued that the shift from a less valued state (i.e., high free-energy) to a more valued state (i.e., low free-energy) is accompanied by positive affect, while a shift in the opposite direction is accompanied by negative affect. Likewise, Ben-Zeev [36] has suggested that emotions are generated when the level of stimulation we have experienced for long enough to get accustomed to it changes, and the change, rather than the general level, is of emotional significance. Accordingly, in the words of the same author, “loss of satisfaction does not produce a neutral state, but misery, and loss of misery does not produce a neutral state either, but happiness” [36].

Similar situations can also be found when people are entertained by magicians or humorists. In both cases, following the surprise elicited by the apparent violation of the physical laws in magic [37] or the incongruity of the situation in humour [38], greatest pleasure is experienced when the trick or the joke is understood. Our suggestion is that pleasure is elicited in the transition from a state of high to low surprise. Critically, magic tricks are performed on a stage where people know that there is no real violation of the physical laws; if such surprising events would happen in everyday life, they would probably be experienced as quite disturbing and unpleasant.

According to our scheme, emotional valence is not estimated itself by the agent but emerges naturally from the process of estimating hidden states by means of free-energy minimization. One could eventually hypothesize that some living organisms, such as humans, explicitly represent valence as one of the causes of their sensations. This means that these agents should also estimate valence (and its uncertainty) like any other hidden state in their generative model. Nevertheless, the explicit representation of valence is not a requirement for emotional valence to exist in our scheme and to play an important role in the adaptation of biological agents to unexpected changes in their world.

To put our valence-based meta-learning scheme to a test, we compared two competing agents in a non-stationary environment. The SPV agent with valence replicated the behaviour of the DP agent that explicitly estimated the volatility of the environment [10]. Nevertheless, the adaptive fitness of the SPV agent to unexpected changes was achieved with the representation of only two hidden states 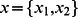 and two parameters 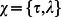, whereas the DP agent required three hidden states 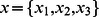 and three parameters 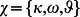. More importantly, the two parameters 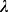 and 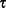 of the SPV agent have a clear psychological interpretation in terms of sensitiveness to emotional valence and mood, respectively. The mood 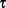 was shown to be important for tracking slow and continuous changes in the environment, known as volatility, whereas the sensitiveness 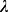 was shown to be crucial for tracking fast and discrete changes, known as unexpected uncertainty. The proposed scheme is very general and does not rely on any particular generative model of how sensory inputs are caused, meaning that it can account for any internal model of the world that defines a particular agent (see [7]).

We also investigated the relationship between estimation uncertainty and *factive* (*happiness* as well as *unhappiness*) and *epistemic* (*hope* and *fear*) emotions. Although psychologists and philosophers have traditionally relied on degrees of belief (uncertainty) in their analyses of these families of emotion [28]–[31], we alternatively relied only on the dynamics of free-energy. In agreement with these more traditional analyses, we found that *epistemic* emotions are indeed more related to higher levels of (estimation) uncertainty than *factive* emotions. However, at the algorithmic level, we reiterate our claim that the computational quantity that unambiguously distinguishes between *factive* and *epistemic* emotions is not degrees of belief, as previously proposed [31], but rather the temporal dynamics of free-energy.

More important for psychological perspectives on emotion, the trajectory invariant representation of emotions in the state space defined by the first and second time-derivatives of free-energy also recapitulates the dimensional view of emotion [39]. Although the first time-derivative of free-energy 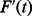 has been intuitively related to the dimension of valence, it is still unclear how to interpret the second time-derivative 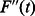 in terms of a psychological construct. The emergence of some forms of emotion, tentatively labelled as *happiness*, *unhappiness*, *hope*, *fear*, *disappointment* and *relief*, also provides support for the notion of basic emotions [40], in the sense that these emotions are exclusively related to very precise dynamics of free-energy. Furthermore, our scheme also encompasses important aspects of cognitive models of emotion [31], [41], [42], in the sense that states of the world (e.g., agents, objects, events), which are relevant for the diversity and complexity of human emotions, can be accounted for within the hierarchical generative model entailed by the agent. To illustrate, happiness (unhappiness) has been related to the negative (positive) first time-derivative and the positive (negative) second time-derivative of the free-energy of some state in the generative model. When the state under consideration is the fate of another person, this can be understood as a specific form of happiness (unhappiness) usually known as ‘joy for another’ (pity) [31].

The concept of value has been largely related to valence in social and affective psychology (see [43]). Our definition of emotional valence in terms of the rate of change of free-energy also provides a formal distinction between valence and value. In the free-energy principle, value is the complement of free-energy in the sense that minimizing free-energy corresponds to maximizing the probability that an agent will visit valuable states, where the evolutionary value of a phenotype is the negative surprise averaged over all the (interoceptive and exteroceptive) sensory states it experiences [2]. This formulation parallels a recently proposed reinforcement learning theory for homeostatic regulation [44], which attempts to integrate reward (valence) maximization with the minimization of departures from homeostasis (free-energy).

Our scheme is also broadly compatible with the predictive coding model of conscious presence [45], which claims that interoceptive inference is the constitutive basis of the *subjective experience of emotions*. Although our formulation treats interoceptive and exteroceptive predictions (and their uncertainty) on an equal footing, one might imagine that prediction of interoceptive states would be a particularly important target for emotional regulation. This is because, from an evolutionary perspective, it is important to maintain a physiological homeostasis and respond adaptively to any unpredicted changes in the internal milieu. Furthermore, the putative emphasis on interoception provides a close link between (literally) ‘gut feelings’ and the computational (inferential) role of emotion that we have described above.

An apparent paradox that might emerge from our definition of emotional valence is related to the common sense notion that both the violation and the fulfilment of expectations can be either positive or negative. As we stated before, according to our scheme, the fulfilment of expectations must always elicit positive emotions whereas the violation of expectations must always elicit negative emotions. Therefore, how can the *subjective experience* of *positive surprises* and *negative expectations* be accounted for within our scheme?

In our perspective, these experiences emerge from a confound between the fulfilment and the violation of expectations across different levels of the hierarchical generative model. To illustrate this, we first need to recall that in the Bayesian brain formulation, agents encode a hierarchical generative model of the causes of their sensations, where states of the world of increasing complexity and abstraction are encoded in higher levels of the hierarchy and sensory data per se are encoded at the lowest level. Let us imagine the case of an old friend who suddenly steps in our door. This unexpected visit can be intuitively related to the experience of a very positive and surprising emotion. However, a more careful analysis can unveil which aspects of this experience are indeed surprising and which are just as expected, given a hierarchical generative model of how sensations are caused. Assuming that our friend has moved to a distant city many years ago, the sudden apparition of this friend certainly violates any expectation about the physical causes of sensations. It would be very surprising to meet a friend at our door when they are expected to be miles away - no matter how beloved they might be. Such a surprising sensation should elicit unpleasantness at the corresponding levels of the model where physical causes of sensations are encoded. Concomitantly, this same sensation should also fulfil more abstract expectations that we might have of being close to beloved ones. The fulfilment of these expectations should conversely elicit pleasantness at higher levels of the generative model where these more abstract causes of sensations are probably represented. With the formalism of a hierarchical generative model, the causes of sensations can be clearly defined and their respective valence properly investigated. In the example above, we would thus consider it more precise to say that ‘we are surprised about the unexpected visit of a friend but happy to be close to a beloved one’. Here, our explanation rests upon the assumption that the *subjective experience of emotion* usually confounds the increasing fulfilment (pleasantness) and violation (unpleasantness) of expectations across different levels of the hierarchical model.

In another example, the reasoning above also can help us to explain how our scheme may account for sensations that are expected but of negative valence (e.g., the expectation of an eminent injury). Let us imagine the case of someone who is walking on the street and suddenly sees a cyclist riding a bicycle dangerously. As the cyclist gets closer, the person becomes increasingly confident that they will be hit by the bicycle. In this situation, the movement of the bicycle fulfils the expectations of the person about how physical bodies should move in the world and, therefore, it elicits pleasantness at those levels of the generative model. Indeed, it would be very surprising (and unpleasant at these levels) if the bicycle suddenly disappeared or made an unexpected movement that violated the physical laws of motion. Nevertheless, the approach of the bicycle also violates other expectations regarding the safety of walking down the street, which are probably represented at different levels of the hierarchical model. At these levels, the approach of the bicycle is very unpleasant and becomes even more unpleasant when the person is indeed injured by the bicycle. Again, in this case, we would consider more precise to say that ‘the person expects the bicycle to hit them - under such environmental conditions - but they do not expect to be injured when walking down the street’.

The flexibility of our scheme to accommodate different generative models may raise some concerns regarding the falsifiability of our theory. However, we would like to clarify that hypotheses derived from our theory should be tested conditional on a particular generative model. Especially given the known diversity of phenotypes in nature, we consider that this flexibility is more a strength than a weakness. Furthermore, generative hierarchical models and free-energy minimization provide a principled way to represent the relationship between hidden states and to understand their dynamics. Nevertheless, further empirical work is still required to better understand at which levels of the hierarchical generative model the violation of expectations might be more closely related to the *subjective experience* of surprise and emotional valence. Our intuition is that the *subjective experience of surprise* is more closely related to violations at lower levels of the hierarchy, whereas the *subjective experience of emotional valence* is more closely related to violations at higher levels.

The distinction between violation and fulfilment of expectations across different levels of the generative model might also help us to further disambiguate the *subjective experience* of other emotions such as *fear* and *anxiety*, which have an important role in psychopathology. One of the ways in which cognitive theories of emotion have distinguished *fear* from *anxiety* is based on the physical and existential aspect of their causes. *Fear* involves threats that are concrete and sudden, whereas *anxiety* is related to threats that are more symbolic, existential and ephemeral [41], [42]. Nevertheless, both *fear* and *anxiety* are related to the prospect of visiting unpleasant states in the future, which in our scheme has been related to a ‘faster and faster’ increase of free-energy over time. To illustrate, let us imagine the case of a spider-phobic person who is presented with a spider. The *subjective experience* of *fear* in this case could be explained as the product of (1) a ‘slower and slower’ increase in the violation of the expectations about the more physical causes of sensations, which encodes the physical recognition of the spider, eliciting unhappiness at these levels; and (2) a ‘faster and faster’ increase in the violation of the expectations about more abstract causes of sensations, such as the increasing probability of being bitten by the spider, eliciting fear at these levels. However, in the case of *anxiety*, there seems to be incongruence between the violation of expectations about the physical and the existential causes of sensations. Therefore, in our perspective, the *subjective experience* of *anxiety* should be expressed as the product of (1) a stationary violation of the expectations about the physical causes of sensations (i.e., the environment is physically perceived as usual) bringing neutrality to these levels, and (2) a ‘faster and faster’ increase in the violation of the expectations about more abstract/existential causes of sensations, eliciting fear at these levels. This incongruence of violation across levels of the generative model could explain the difficulty that anxious people have to attribute concrete causes to their fears.

Our formulation of emotional valence might also be of importance in the investigation of affective and other mental disorders, such as depressive and anxiety disorders [46]. For instance, when we use our model to explain major depressive disorder (MDD), which is a complex debilitating psychiatric condition that is largely characterized by persistent low mood and decreased interest or pleasure in usually enjoyable activities [47], we immediately find the crucial role played by our mood model parameter 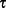. In our meta-learning scheme, when mood is low (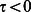), the estimation uncertainty of environmental states is overestimated and top-down predictions become under confident. Theoretical computational simulations has shown that pathological under confidence in top-down predictions can impair behaviour due to a failure in eliciting sufficient sensory prediction errors [48]. Consequently, the agent reacts less vigorously toward, or away from stimuli that might have been previously evaluated as pleasant or unpleasant. In fact, several studies have reported that clinically depressed individuals spend significantly more time looking at negative stimuli [49]–[52]. A subsequent, and cyclical, increase in mood (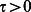) could eventually explain manic episodes in bipolar disorders [53]. Manic episodes are characterized by a distinct period during which patients experience abnormally and persistently elevated, expansive, or irritable mood [54]. In fact, a pathological increase in the precision of top-down predictions has also been shown to induce perseverative behaviours [48]. It would be interesting to investigate how mood induction in healthy subjects might affect their performance on tasks where tracking volatility is necessary. According to our theory, we would predict that subjects with mood levels below and above the optimum for tracking some particular level of environmental volatility should benefit from positive and negative mood induction, respectively. More precisely, an inverted U-shaped performance curve is predicted with depressed and manic patients found at the lowest and highest extremes of the mood range.

A reasonable approach to test hypotheses derived from our theory would be to invert a generative model (i.e., estimate the unknown model parameters) for the experimental task at hand using variational Bayes [55]. The free-energy computed during this inversion process can then be exploited to estimate the emotions at different levels of the hierarchical generative model according to our scheme. A complete characterization of the generative model could eventually be relaxed if a direct measure of the free-energy or, under simplifying assumptions, prediction error is also available. Indeed, the quantity that matters for testing our emotional valence hypothesis is the rate of change of free-energy rather than the generative model itself.

Future empirical work should investigate the correlation between the estimated emotional valence (i.e., the first time-derivative of free-energy) and verbal-reports of valence for a variety of experimental conditions. As previously mentioned, free-energy is an upper bound on surprise and its minimization also entails prediction error reduction. In this perspective, recording prediction error signals in the brain, computing their temporal derivatives and correlating them to verbal-reports of valence could be a suitable procedure. Human neuroimaging studies have shown that the orbitofrontal cortex plays an important role in linking different types of reward to hedonic experience (see [56]). Orchestrated with the striatum [57], which has been traditionally implicated in reward prediction error [58] and saliency [59], those two regions might be of particular relevance to the investigation of our scheme in the brain. In biologically plausible implementations of free energy minimisation, precision (i.e., the inverse of uncertainty) is encoded by the gain of cells reporting prediction error [2]. This directly implicates the classical ascending neuromodulatory transmitter systems like dopamine, acetylcholine and norepinephrine in the encoding of uncertainty. The diverse and complex interactions between these neurotransmitters and their role in encoding different forms of uncertainty are still far from being clearly understood [11], [60], [61]. Future work will address how our meta-learning scheme, which links the rate of change of free-energy (prediction error) to estimation uncertainty (precision), can help in elucidating the complex interaction between these neurotransmitters and the activity in their target brain areas.

To conclude, by providing a general framework in which different perspectives on emotion can be formally interrelated, and by demonstrating how emotional valence can dynamically regulate uncertainty, we hope to contribute to paving the way for future computational studies of emotion in learning and uncertainty.
